# Assessment of nursing managers’ awareness and hospital preparedness for disasters: a cross-sectional study

**DOI:** 10.1186/s12873-024-01122-7

**Published:** 2024-10-25

**Authors:** Borhan Rahimi, Arezoo Yari, Fatemeh Rafiei, Mokhtar Mahmoudi

**Affiliations:** 1 Master’s Student in Emergency Nursing, Health Development Research Institute, School of Nursing and Midwifery, Kurdistan University of Medical Sciences, Sanandaj, Iran; 2 Social Determinants of Health Research Center, Health Development Research Institute, Kurdistan University of Medical Sciences, Sanandaj, Iran; 3 Department of Health in Emergencies and Disasters, School of Medicine, Kurdistan University of Medical Sciences, Sanandaj, Iran; 4 Department of Epidemiology and Biostatistics, School of Public Health, Tehran University of Medical Sciences, Tehran, Iran; 5 Clinical Care Research Center, Health Development Research Institute, School of Nursing and Midwifery, Kurdistan University of Medical Sciences, Pasdaran Street, Room 1, Sanandaj, Iran

**Keywords:** Awareness, Disaster planning, Disasters

## Abstract

**Introduction:**

Preparedness, focused on planning, training, and research, is one of the primary stages of the disaster management cycle. Accordingly, this study was conducted to determine the level of awareness in nursing managers and the preparedness of hospitals for disasters in the hospitals of Sanandaj, the capital of Kurdistan Province.

**Methods:**

This cross-sectional study was conducted in 2023, with a total of 167 Nursing Managers in Sanandaj selected as the research sample using a census approach. Data were collected using a demographic information form, WHO Hospital Emergency Response Checklist, and managers’ emergency awareness questionnaire. Data were analyzed using Chi-square tests, Fisher’s exact test, independent t-tests, analysis of variance (ANOVA), and Pearson correlation. Data analysis was performed using SPSS v26 (*P* < 0.05).

**Findings:**

The results indicated that the overall mean score of managers’ awareness was 77.89%, categorized as good. The assessment of hospital preparedness showed that the overall emergency preparedness level of hospitals in Sanandaj was 69.23%, considered strong. Among the dimensions of hospital preparedness, the highest score was in the command-and-control dimension at 83.33%, while the lowest was in the human resources dimension at 56.66%.

**Conclusion:**

The findings indicated a high level of awareness among nursing managers and a strong level of hospital preparedness in Sanandaj. However, improving and enhancing specific dimensions may require targeted educational and organizational approaches.

## Introduction

Today’s world is faced with an increasing frequency of disasters and emergencies. Disasters and emergencies significantly impact public health, general well-being, and the welfare of affected populations [1]. The Center for Research on the Epidemiology of Disasters (CRED) reported a total of 399 natural disasters in 2023, which affected 93.1 million people, resulted in 86,473 fatalities, and caused economic losses valued at approximately $202.7 billion [2].

Disasters can be broadly categorized into natural events, such as earthquakes, floods, and storms, and human-made incidents, including military conflicts, human-induced biological events, and radioactive material leaks [3]. The occurrence of most of natural disasters is inevitable, and they affect communities worldwide [4]. Given the widespread occurrence of emergencies and natural disasters, managing their impacts—particularly health-related consequences—is essential, as supported by international frameworks like the “Sendai Framework for Disaster Risk Reduction 2015–2030” (SFDRR) [5].

Preparedness, which focuses on planning, education, and research, is a critical stage in disaster management. It has long been a concern and has been increasingly emphasized as an integral part of sustainable development in societies, highlighting the importance of activities necessary to achieve a high level of preparedness [6]. Preparedness is considered both a result and a goal of disaster risk reduction activities. The health system, as a fundamental pillar of society, plays a crucial role in disaster risk reduction. Therefore, ensuring the preparedness of the health system is vital to the successful implementation of SFDRR [7]. Regardless of the cause, disasters and emergencies necessitate the preparedness of healthcare facilities to reduce mortality and injury in the community. In many natural disasters, hospitals are directly affected, suffering structural and non-structural damage, as well as disruptions in equipment, personnel, and even organizational and managerial functions, rendering them unable to provide services to disaster victims [8].

The role and importance of hospitals in critical events are crucial for saving lives [9], and their preparedness is a key factor in disaster management [10]. Enhancing hospitals’ ability to manage extraordinary situations is among the top priorities in mitigating the adverse effects of natural disasters [11]. Although public Education and awareness raising is one of the the adaptive behaviors for combating specific hazard [12], Additionally, the preparedness and awareness of managers in healthcare facilities, coupled with the necessary knowledge of disaster management, enable them to plan effectively and respond wisely during emergencies, fulfilling their roles as required [13]. These managers must use their skills and quick decision-making to provide the necessary care, preventing the exacerbation of problems and the development of complications [14].

Nurses, as the largest group of healthcare personnel [15] and accounting for approximately 56% of hospital staff [16], are key members of crisis management teams and play a crucial role in all stages of disaster management [17]. The effectiveness and efficiency of nurses depend on the use of appropriate management methods and techniques by head nurses and nursing managers [18]. The management of this group of healthcare providers is the responsibility of nursing managers, who operate at various levels, including managing different hospital departments, middle and strategic management, determining the organization’s mission, and enhancing the perspectives and values of employees and nurses [19, 20]. Several factors influence successful management, especially during disasters. For instance, a study demonstrated a positive correlation between higher managerial positions and increased confidence and disaster preparedness [21]. Similarly, another study found that male nurses who were married, held permanent employment, and had higher education levels were significantly more prepared [22]. Additionally, A recent study highlighted the gaps in disaster preparedness and the core competencies of emergency nurses, which need to be addressed [23].

According to the latest national census in Iran, Kurdistan Province, located in western Iran, covers an area of 29,137 square kilometers with a population of 1,603,011, accounting for approximately 1.78% of the country’s area and 2.01% of its population. Sanandaj, the capital of Kurdistan Province, has a population of 501,402 [24]. The province’s topography, geographic and ethnic diversity, and border location make it susceptible to various natural and human-made disasters [25–28]. Given the disaster-prone nature of Kurdistan Province, particularly Sanandaj [29], and the region’s low socio-economic indicators [25], as well as the varying results from studies assessing hospital and nursing managers’ preparedness across Iran, it is crucial to ensure the continuity and resilience of hospitals in providing healthcare services during disasters. Furthermore, it is essential to inform healthcare managers and policymakers about the level of preparedness of hospitals and nursing managers for disasters. Consequently, this study was carried out to assess the awareness of nursing managers and hospital preparedness for disasters and emergencies in Sanandaj.

### Methodology

This cross-sectional study was conducted in 2023. The research population included hospitals in Sanandaj, Kurdistan Province, namely Be’sat, Tamin-e Ejtemaei, Tohid, Dr. SeyedoShohadaei, Qods, and Kosar hospitals, along with the nursing managers employed in these hospitals using a census approach. Among these, four hospitals were under the supervision of the Ministry of Health, one hospital was managed by the Social Security Organization, and one hospital was privately owned. In this study, nursing managers were defined as individuals holding various organizational titles responsible for managing nurses. These titles included chief nursing officer (matron), director of nursing (supervisor), head nurse, and subunit manager. The inclusion criteria for nursing managers in the study were: (1) holding an official nursing management position, (2) having at least one year of experience as a nursing manager, and (3) willingness to participate in the study. The exclusion criterion was incomplete questionnaire responses.

### Methods

Data were collected using three questionnaires. The first was a demographic information form for nursing managers, which included details such as the hospital of employment, organizational position, service unit, gender, age, education, and managerial or supervisory experience.

The second research tool was the national standard checklist, developed by a group of experts in disaster and emergency health at the Deputy of Treatment, Ministry of Health, Treatment, and Medical Education in Iran as part of the National Hospital Preparedness Program. This checklist is used for hospital accreditation and was completed through observation and interviews with hospital heads. If further information was required on specialized areas of the checklist, input was sought from relevant experts with the coordination of the hospital’s emergency management officer. The 91-item checklist is one of the national tools for assessing health in disasters and is known as “Tool No. 5: Hospital and Healthcare Center Disaster Preparedness Assessment Tool.” It was introduced in 2011 by the World Health Organization’s Regional Office for Europe to assist hospital staff and emergency managers in providing effective responses to the most likely disaster scenarios [30]. The checklist comprises nine dimensions and a total of 91 questions that assess hospital preparedness. Additionally, it includes 11 questions on hospital characteristics, such as hospital name, type, number of approved and active beds, number of ICU beds, number of isolation rooms, annual hospital admissions, total staff, number of ambulances, trained crisis management administrators, and the year the hospital was established. The checklist dimensions include: command and control, communication, safety and security, triage, surge capacity, continuity of essential services, human resource, Logistics and supply management, and post-disaster recovery. Responses to these questions were categorized as follows: (a) due to review (1 point), (b) in progress (2 points), and (c) completed (3 points). Each of the nine sections had defined ranges for weak, moderate, and strong scores. However, after aggregating the scores, the overall hospital preparedness was classified into one of the following categories: weak preparedness (93–152), moderate preparedness (153–212), and strong preparedness (213–273) [30].

The third questionnaire focused on assessing the awareness of managers concerning disasters and emergencies. This questionnaire was developed based on national guidelines in Iran and has been validated in terms of reliability and validity by Amiri et al. (2013) and Hekmatkhah et al. (2012) [13, 31]. All study participants completed the awareness assessment questionnaire. The questionnaire consists of 33 questions that evaluate the managers’ awareness regarding hospital preparedness and disaster management, along with an additional seven demographic questions. Each question had three possible responses: Yes (2 points), Somewhat (1 point), and No (0 points). The manager awareness questionnaire was structured on a Likert scale, with final scores categorized as follows: a mean score of less than 50% (0–32) indicated poor awareness, a score between 50% and 75% (33–49) indicated moderate awareness, and a score above 75% (50–66) indicated good awareness.

After obtaining a letter of recommendation from Kurdistan University of Medical Sciences, the researcher visited the hospitals under study across all three work shifts. The standard hospital preparedness checklist and the manager awareness questionnaire were distributed among the research sample within the study population. The researcher provided initial instructions on how to complete the questionnaire, and the questionnaires were completed in the presence of the researcher. The hospital preparedness questionnaire was given to hospital heads, while the manager awareness questionnaire was distributed to the chief nursing officers (matrons), supervisors, head nurses, and subunit managers. Clarifications were provided if any questions or ambiguities arose.

### Data analysis

The collected data were coded analyzed using SPSS v26. The normality of the data was evaluated using Kolmogorov-Smirnov test, also based on the Central Limit Theorem, with a sample size of 167 participants, the sampling distribution of the mean approximates normality. Therefore, the assumption of normality for parametric tests is reasonably justified. Additionally, the homogeneity of variances was tested using Levene’s test, and the results indicated that the variances were equal across groups, satisfying the assumption of homogeneity necessary for valid interpretation of the parametric tests. Descriptive statistics were used to present quantitative variables as mean ± SD, and qualitative variables with frequencies and percentages. Inferential statistics were employed to examine the relationships between qualitative variables using the Chi-square test and Fisher’s exact test. To compare the means of quantitative variables across different levels of qualitative variables, independent t-tests and ANOVA were used. Pearson correlation tests were also conducted to assess the relationships between quantitative variables (*P* < 0.05).

### Findings

Sanandaj has eight hospitals, Head of Aria hospital refused to participate and a military hospital was not included in the study, six hospital heads agreed to participate and they also answered The 91-item checklist for their hospital then percentage of participation was 85.71% in Hospitals preparedness Assessment. The percentage of participation in assessing the awareness of managers was 94.88% as five managers did not meet the inclusion criteria, nine of them did not participate, 167 nursing managers agreed to participate and answered the awareness assessment questionnaire. Ultimately, 167 nursing managers from six hospitals in Sanandaj were participated in the study. The analysis of demographic data revealed that the majority of the participants were female (69.5%), with an average age of 41.92 ± 5.89 years. Most participants held a bachelor’s degree (79.6%), and head nurses constituted the largest group among the participants (51.5%). The demographic characteristics of the study subjects, along with the results, are presented in Table 1.

**Table 1:** Demographics of nursing managers

Variable		Organizational rank of mangers and hospitals	%	Frequency
Hospital	A		29.3	49
	B		28.1	47
	C		18	30
	D		7.2	12
	E		5.4	9
	F		12	20
Position	Subunit Heads		10.2	17
	Head nurse		51.5	86
	Supervisor		34.7	58
	Matron		3.6	6
Ward	Quality improvement		3	5
	Adults internal-surgery wards		17.4	29
	Neonatal and pediatrics internal-surgery wards		4.8	8
	Adults ICU		7.2	12
	Neonatal and pediatrics ICU		2.4	4
	Emergency		9	15
	Nursing office		38.9	65
	Clinics		4.8	8
	Gynecology and obstetrics		3	5
	Operation room, angiography and electroshock		4.8	8
	Psychiatric nursing		4.8	8
Gender	M		30.5	51
	F		69.5	116
Education	Bachelors’ degree		79.6	133
	Masters’ degree		19.2	32
	PhD		1.2	2
			SD	Mean
Age			5.89	41.92
Management record			6.34	8.28

The findings indicated that the average awareness score of nursing managers was 77.89% ± 14.22, reflecting a high level of awareness among nursing managers regarding disasters and emergencies. The results also revealed that the awareness levels of most managers—66.5% or 111 individuals—were categorized as good, 29.9% or 50 individuals had moderate awareness, and 3.6% or 6 individuals had low awareness (Table 2).

**Table 2:** Mean and SD of managers’ awareness

Awareness	Min	Max	Mean	SD
Score	15	66	51.41	9.39
%	22.73	100	77.89	14.23

Table 3 shows that the highest average awareness score of managers regarding disasters and emergencies was in the organizational dimension, with a mean of 53.10, while the lowest average was 48.16. Regarding awareness based on organizational position, the highest average score was observed among chief nursing officers (Matron) with a mean of 54.16, while the lowest awareness was among subunit heads with an average of 44.29. In terms of hospital departments, the highest awareness was found in the pediatric and neonatal units, with a mean score of 57, while the emergency departments had the lowest, with a mean of 39.46. Additionally, the highest awareness was observed among female nurses, with a mean score of 52, and those with a doctoral degree, with an average of 52.5 (Table 3).

**Table 3:** Comparison of mean and SD of managers’ awareness scores based on hospital and demographic characteristics of managers

			SD		Mean		Test			
							**Statistics**		Sig.	
Hospital		A	9.72		53.04		F = 1.099		0.363	
		B	8.74		49.57					
		C	8.87		51.96					
		D	8.72		48.16					
		E	15.25		50.88					
		F	7.74		53.10					
Position		Subunit Heads	11.11		44.29		F = 4.336		0.06	
		Head nurse	8.81		52.82					
		Supervisor	9.18		51.12					
		Matron	6.21		54.16					
Ward		Quality improvement	2.38		52.8		F = 4.069		0.0001	
		Adults internal-surgery wards	6.59		53.86					
		Neonatal and pediatrics internal-surgery wards	5.6		57					
		Adults ICU	6.93		55.83					
		Neonatal and pediatrics ICU	11.11		54.25					
		Emergency	11.96		39.46					
		Nursing office	9.34		51.04					
		Clinics	7.66		48.12					
		Gynecology and obstetrics	7.96		53					
		Operation room, angiography and electroshock	9.56		54.37					
		Psychiatric nursing	7.26		52.75					
Gender		M	9.91		50.07		T=-1.219		0.225	
		F	9.14		52					
Education		Bachelors’ degree	9.78		51.18		F = 0.199		0.820	
		Masters’ degree	8.01		52.31					
		PhD	0.70		52.5					
							**Pearson’s correlation**		Sig.	
Age							-0.007		0.926	
Management record							0.013		0.863	

The results presented in Table 4 reveal that the largest hospital in the study had 540 registered beds, while the smallest had 50 beds. The overall average preparedness of the hospitals was 69.23%, categorized as strong. However, the findings also indicated that despite the overall strong preparedness, the hospitals under the Ministry of Health’s supervision showed a moderate level of preparedness for disaster response. An analysis of the nine dimensions of preparedness showed that the highest level of preparedness was in the command-and-control dimension, with an average of 83.33%, while the lowest level of preparedness was in the human resource dimension, with an average of 56.66%. (Table 4)

**Table 4:** Assessment of hospital preparedness levels based on the nine dimensions

Dimensions	Min (%)	Max (%)	Mean (%)	SD (%)
Command and control	50	100	83.33	17. 48
Communication	27.77	100	62.96	28.25
Safety and security	45.45	90.90	67.42	17.81
Triage	45	90	78.33	17.51
Surge capacity	46.15	100	68.58	20.12
Continuity of essential services	31.25	100	75	23.38
Human resources	10	86.66	56.66	29.81
Logistics and supply management	40	80	64.16	13.57
Post-disaster recovery	44.44	100	74.99	19.16
Total score of preparedness	36.81	95.60	69.23	19.97

## Discussion

The primary objective of this study was to determine the awareness of nursing managers and the preparedness of hospitals in Sanandaj for disasters and emergencies. The overall awareness score of nursing managers in these hospitals was at a good level. Specifically, the highest levels of awareness were observed among matrons and nurses in pediatric and neonatal units. Furthermore, female nurses and those with higher educational qualifications demonstrated greater awareness. These results are consistent with Mohammadi et al. (2024), who reported that the awareness of participating nursing managers was at a good level [32]. In contrast, Danialipour et al. (2022) found that nurses had a low level of disaster preparedness knowledge and an overall moderate level of disaster preparedness [22]. Similarly, Jahani et al. (2019) reported that the average awareness of hospital managers, including nursing managers, was at a moderate level [33]. Lin et al. (2023) also found that while disaster preparedness among nurses was low, it was moderate among participating nursing managers [34]. Additionally, Reedy et al. (2022) assessed nurse leader confidence in emergency management and disaster preparedness as moderate [21].

A review of the literature on managerial awareness of disasters and emergencies reveals mixed results. The discrepancies in these findings could be attributed to the timing of the studies, as more recent studies tend to show higher awareness among unit managers. This increase in awareness may be due to better familiarity with accreditation standards and enhanced disaster awareness training, a trend confirmed by the current study, where hospital managers’ awareness of disasters was comparable to the findings of Mohammadi et al. Another reason for discrepancies in these findings could be differences of the target populations.

In the present study, the matrons had the highest level of awareness, while subunit heads had the lowest. Additionally, the highest levels of preparedness were observed among managers of pediatric and surgical units, while the lowest awareness levels were found among emergency department managers. These findings align with the studies by Jahani et al. (2019) and Reedy et al. (2022), who reported that after nursing managers (matrons), supervisors, and head nurses had the highest awareness levels [21, 33]. However, these results differ from Lin et al. (2022), Tzeng et al. (2016), and Nilsson et al. (2016), which found that nurses working in emergency and critical care units had higher disaster preparedness [34].

The differences in study results could be attributed to the structure of emergency departments and the variations in hospital care models between Iran and other countries. The high patient load and shortage of nurses in Iranian hospital emergency departments, particularly in Kurdistan province, often prevent managers from regularly attending disaster and crisis management conferences. This is concerning, as emergency department managers, being on the frontline, should have a higher level of preparedness for disasters. According to the findings of this study, the lack of preparedness among emergency department managers could serve as a warning to hospital administrators about their ability to respond effectively to disasters. It is recommended that these managers participate more actively in training programs, annual drills, and crisis and disaster congresses to enhance their preparedness.

The present study showed that the overall preparedness of hospitals in Sanandaj was classified as strong in qualitative terms. The highest preparedness was observed in the command-and-control dimension, while the lowest was in the human resources dimension. Despite the strong preparedness of hospitals in Sanandaj, the overall preparedness of hospitals under the Ministry of Health was at a moderate level. This finding aligns with the studies by Jahani and Mohammadi, which also reported moderate preparedness among hospitals under the Ministry of Health in southeastern Iran and western Guilan [32, 33]. Sheikhbardsiri et al. (2021) confirmed this finding, reporting that the responsiveness of six hospitals in Kerman was at a moderate level. Their study also showed that the highest preparedness was in the command-and-control dimension, while the human resources dimension, similar to the present study, was classified among the weakest aspects [35].

The focus on the human resources dimension as one of the weakest areas of hospital preparedness in both studies should be a point of concern for hospital administrators at the provincial and national levels. The more skilled and capable the human resources in healthcare systems like hospitals, the better the overall preparedness for disasters. In Jannati’s (2018) study, the preparedness of hospitals in Tabriz, assessed using a Ministry of Health-approved questionnaire, was reported as moderate, with the highest preparedness in communications and the lowest in surge capacity [36]. These findings differ from those of the present study, where fewer hospitals were assessed, potentially a limitation of this study. In the present study, communications preparedness was moderate, while surge capacity preparedness was strong. Meanwhile, Baki Hashemi et al. (2022) indicated that the relative preparedness for disasters in Guilan hospitals was moderate, and the same level of preparedness was found in hospitals under the Ministry of Health [37]. Blanchette et al. (2023) also reported that hospital preparedness in the Netherlands was moderate, which contrasts with the findings of the present study [38]. However, in most reviewed studies, the command-and-control dimension had the highest mean score, consistent with the present study. The lowest mean score was often found in the logistics and supply dimension, which differs in some aspects from the present study; however, the human resources dimension was also identified as a weak area, aligning with the current findings. The differences in study results could also be due to the use of different tools to assess hospital disaster preparedness. In this study, the standard Ministry of Health checklist was used to assess the nine dimensions of hospital preparedness, a tool that is recognized as valid and reliable in accreditation evaluations. However, other studies reviewed used different tools.

## Conclusion

The findings of the present study indicated that hospital managers in Sanandaj had a good level of awareness regarding disasters and emergencies, with the highest awareness observed in the pediatric and neonatal departments, and the lowest in the emergency departments. Additionally, the study showed that the preparedness of hospitals in Sanandaj for disasters and emergencies was strong. An analysis of the nine dimensions of preparedness revealed that the highest readiness was in the command-and-control dimension, while the lowest was in human resource management. Sanandaj, with its high population density and significant vulnerability to disasters, requires continuous improvement in managerial awareness and hospital preparedness. Similar to many studies conducted in this field, managerial awareness, particularly in emergency departments, and the shortage of human resources as a critical weakness in disaster preparedness in Sanandaj, should be prioritized. The primary limitation of this study was the difficulty in accessing hospital officials to complete the standard hospital preparedness checklist. To overcome this challenge, the researcher had to visit the hospitals multiple times, directly meet with the officials, and encourage them to complete the checklist. The second limitation was the participation of a small number of hospitals in the study. Although there are only six public and private hospitals in Sanandaj, and the researcher made every effort to sample all of them, it is recommended that future studies include all 20 hospitals in Kurdistan province to assess the overall preparedness of hospitals and their managers’ awareness across the region.

## Data Availability

The data that support the findings of this study are not openly available due to reasons of sensitivity and are available from the corresponding author upon reasonable request. Data are located in controlled access data storage at Kurdistan University of Medical Sciences. From Corresponding Author Email: mokhtar.mahmoudi@muk.ac.ir.
